# Growth hormone deficiency with late-onset hypothalamic hypoadrenocorticism associated with respiratory and renal dysfunction: a case report

**DOI:** 10.1186/s12902-020-0536-3

**Published:** 2020-04-16

**Authors:** Nami Kojima, Nobuyuki Koriyama, Akinori Tokito, Kazuma Ogiso, Koshi Kusumoto, Satoshi Kubo, Yoshihiko Nishio

**Affiliations:** 1 Department of Diabetes and Endocrine Medicine, National Hospital Organization Kagoshima Medical Center, 8-1 Shiroyama-cho, Kagoshima, 892-0853 Japan; 2 Department of Diabetes and Endocrine Medicine, Kagoshima University Graduate School of Medicine and Dental Sciences, Kagoshima University, 8-35-1 Sakuragaoka, Kagoshima, 890-8520 Japan

**Keywords:** Growth hormone (GH), Childhood-onset growth hormone deficiency (GHD), Empty Sella turcica, Late-onset secondary hypoadrenocorticism, Panhypopituitarism, Restricted ventilation disorder, Estimated glomerular filtration rate (eGFR)

## Abstract

**Background:**

The prevalence of childhood-onset growth hormone (GH) deficiency (GHD) is estimated to be approximately 1 in 5000 or more, with the cause unknown in most cases (idiopathic isolated GHD). However, additional disorders of secretion of other pituitary hormones reportedly develop over time, with a frequency of 2–94% (median, 16%). Furthermore, median times to development of other anterior pituitary hormone deficiencies have been reported to be 6.4–9.4 years. On the other hand, adult patients affected by childhood-onset GHD reportedly develop impaired ventilation function due to reduced lung volumes and respiratory pressures, probably due to reductions in respiratory muscle strength. In addition, GH is known to play a role in stimulating the glomerular filtration rate (GFR), and the estimated GFR (eGFR) is decreased in patients with GHD.

**Case presentation:**

This case involved a 65-year-old woman. Her short stature had been identified at around 3 years of age, but no effective treatments had been provided. The patient was mostly amenorrheic, and hair loss became apparent in her late 30s. She developed hyperuricemia, dyslipidemia, and hypertension at 45 years of age. In addition, the patient was diagnosed with hypothyroidism at 50 years of age. At 58 years of age, endocrinological examination showed impaired secretion of thyroid-stimulating hormone, luteinizing hormone/follicle-stimulating hormone, and growth hormone, and magnetic resonance imaging showed an empty sella turcica. However, secretion ability of adrenocorticotropic hormone was retained. At 63 years of age, respiratory function tests confirmed a markedly restricted ventilation disorder (vital capacity, 0.54 L; percentage predicted vital capacity, 26.9%). Renal function had also decreased (eGFR, 25.0 mL/min/1.73 m^2^). Furthermore, she was diagnosed with hypothalamic secondary hypoadrenocorticism. The patient developed CO_2_ narcosis at 65 years of age, and noninvasive positive pressure ventilation was started.

**Conclusions:**

The rare case of a 65-year-old woman with childhood-onset GHD with panhypopituitarism, including late-onset secondary hypoadrenocorticism in her 60s, associated with severely impaired respiratory function and renal dysfunction, was reported. In GHD patients with risk factors for progression from isolated GHD to combined pituitary hormone deficiency, such as empty sella turcica, lifelong endocrinological monitoring may be important.

## Background

The prevalence of childhood-onset growth hormone (GH) deficiency (GHD) is estimated to be approximately 1 in 5000 or more, with the cause unknown in most cases (idiopathic isolated GHD) [1]. However, additional disorders of secretion of other pituitary hormones reportedly develop over time, with a frequency of 2–94% (median, 16%) [2]. Furthermore, median times to development of luteinizing hormone (LH)/follicle-stimulating hormone (FSH), adrenocorticotropic hormone (ACTH), and thyroid-stimulating hormone (TSH) deficiencies have been reported to be 6.4–9.4 years [2].

On the other hand, hormones are well known to regulate and/or affect skeletal muscle contractility, energy supply and metabolic pathways, membrane permeability, and protein turnover due to a combination of different mechanisms. Respiratory muscle has the physiological and biochemical characteristics of skeletal muscle. Various hormone disturbances are therefore associated with impairment of respiratory muscle function [3]. For example, adult patients affected by childhood-onset GHD reportedly develop impaired ventilatory function due to reduced lung volumes and respiratory pressures, probably due to reductions in respiratory muscle strength [4]. In addition, GH is known to play a role in stimulating the glomerular filtration rate (GFR) [5, 6], and GFR is decreased in patients with GHD [7].

The rare case of a 65-year-old woman with childhood-onset GHD with panhypopituitarism, including late-onset secondary hypoadrenocorticism appearing in her 60s, is presented. The patient showed impairment of both respiratory and renal functions.

## Case presentation

**Fig. 1:**
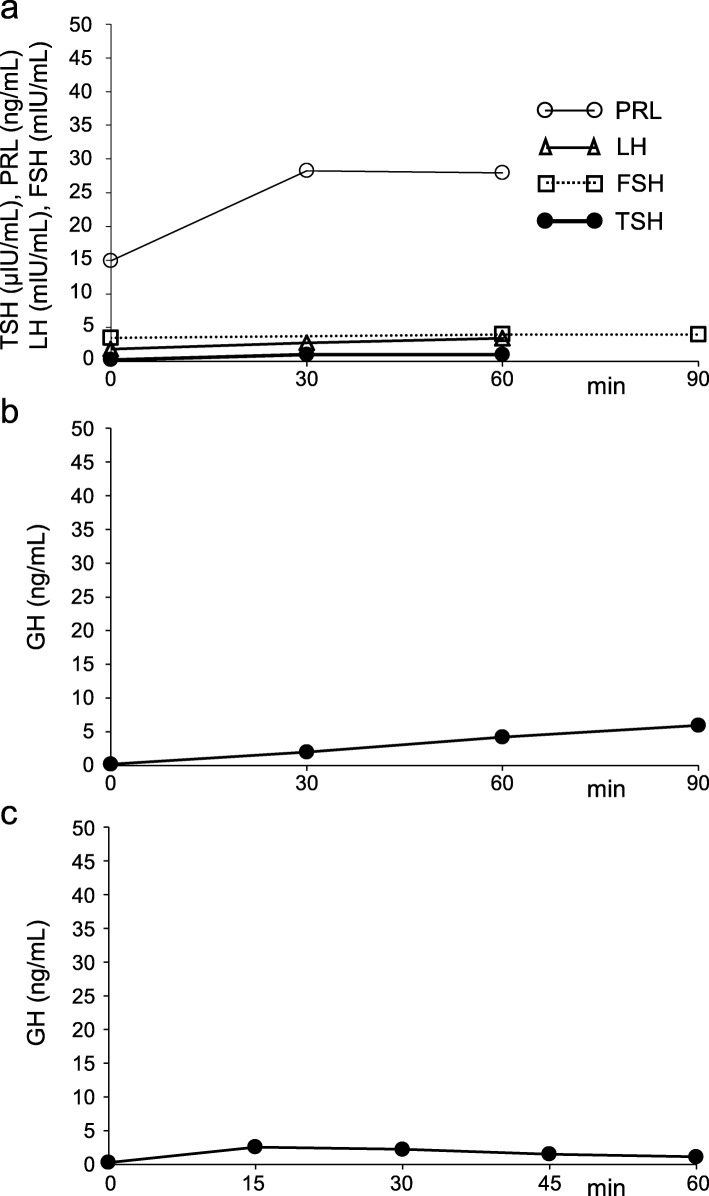
Findings of endocrine function tests 1. Findings at 58 years of age. TSH (closed circles, thick solid line) and PRL (open circles, thin solid line) responses to TRH (500 μg, i.v.), and LH (open triangles, thick solid line) and FSH (open squares, dashed line) responses to LH-RH (100 μg, i.v.) (**a**). GH response to arginine (0.5 g/kg, d.i.v.) (**b**). GH response to GHRP-2 (100 μg, i.v.) (**c**). TSH, thyroid-stimulating hormone; PRL, prolactin; TRH, thyrotropin-releasing hormone; LH, luteinizing hormone; FSH, follicle-stimulating hormone; LH-RH, luteinizing hormone-releasing hormone; GH, growth hormone; GHRP-2, GH-releasing peptide-2; i.v., intravenous infusion; d.i.v., drip intravenous infusion

**Fig. 2:**
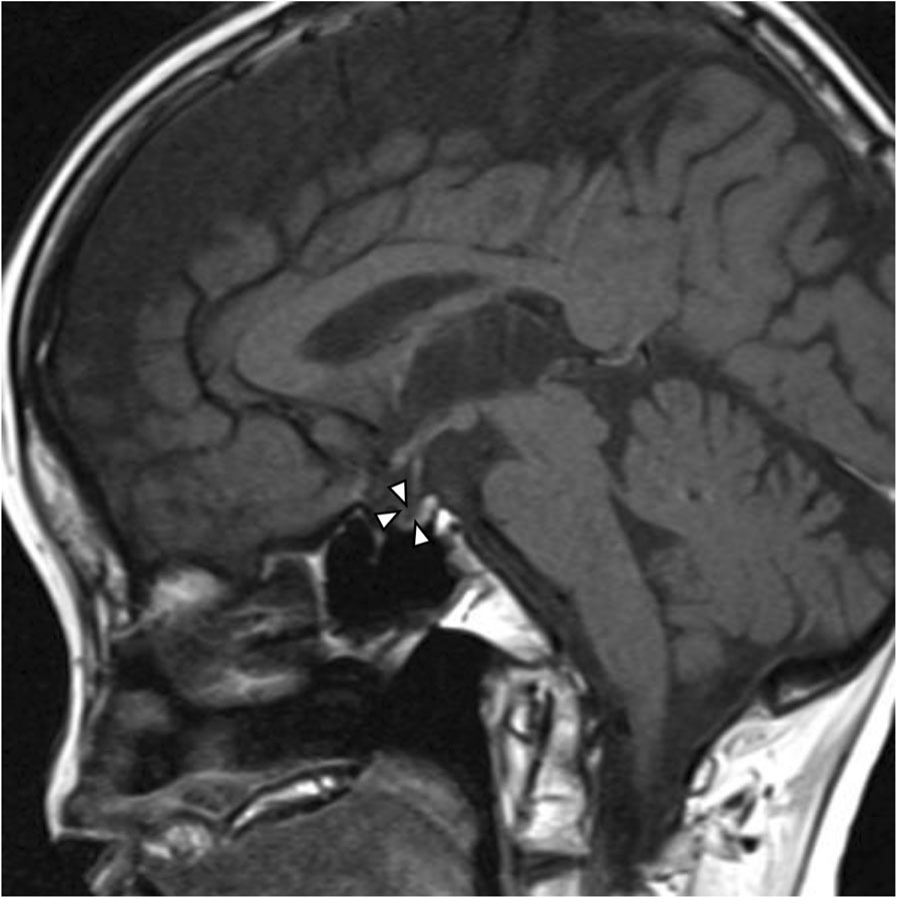
Hypothalamic-pituitary MRI image. A sagittal T1-weighted image is shown, confirming an empty sella turcica. No abnormality in the hypothalamus-pituitary stalk is evident, and a high-intensity signal is present in the posterior lobe. The anterior lobe is atrophic. Arrowheads indicate an empty sella turcica

**Fig. 3:**
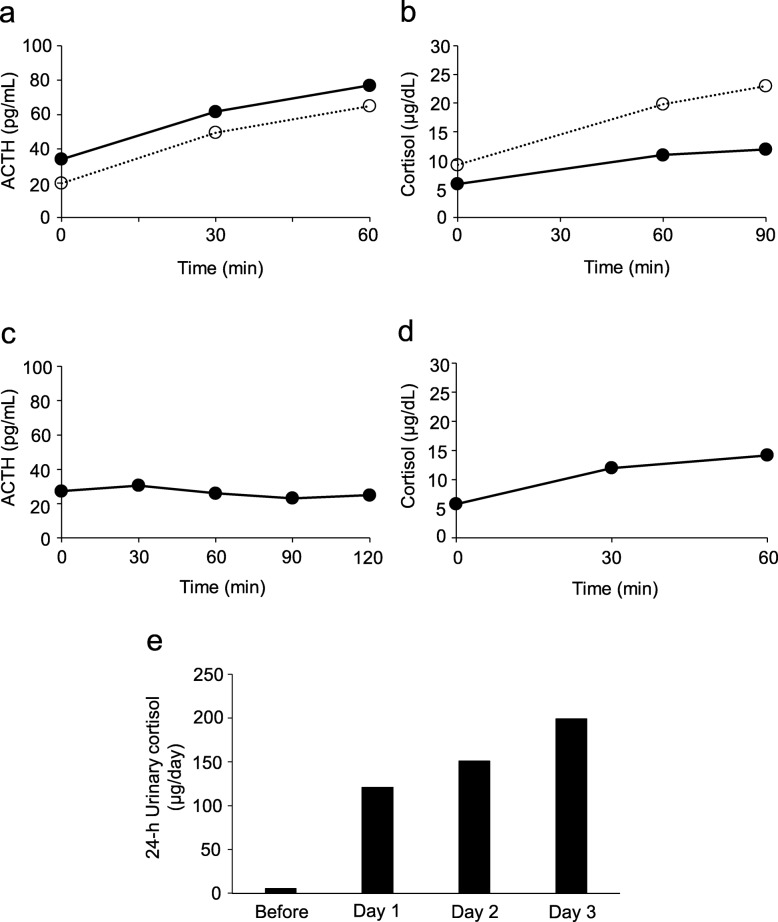
Findings of endocrine function tests 2. (**a**) ACTH and (**b**) cortisol responses to a CRH (100 μg, i.v.**)** stimulation test. In both cases, a closed circle (solid line) indicates the value at 63 years of age, compared to results at 58 years of age (open circles and dashed lines). (**c**) Insulin tolerance test. ACTH response to insulin (0.05 units/kg bodyweight, i.v.). (**d**) Rapid ACTH stimulation test. Cortisol response to ACTH **(**tetracosactide acetate 0.28 mg, i.v.**)**. (**e**) Prolonged ACTH simulation test. Cortisol response to ACTH **(**tetracosactide acetate, 0.56 mg, i.m.**)**. **c**, **d**, and **e** all show data at 63 years of age. ACTH, adrenocorticotropic hormone; CRH, corticotropin-releasing hormone; i.v., intravenous; i.m., intramuscular

The patient was a 65-year-old woman with no relevant family history. She was born without any perinatal anomalies, although short stature was identified at around 3 years of age, but she never received effective treatments. Withdrawal bleeding occurred from around the age of 16 years after hormone-replacement therapy was started to address the absence of secondary sexual characteristics and primary amenorrhea. However, treatment was self-interrupted 1 year later, and she remained amenorrheic thereafter. The patient noticed hair loss in her late 30s. She visited a medical practitioner due to gout at 45 years of age and was subsequently treated for hyperuricemia, dyslipidemia, and hypertension at the clinic. In addition, the patient was diagnosed with hypothyroidism at 50 years of age, and thyroid hormone-replacement therapy was started (levothyroxine sodium hydrate, 50 μg/day). In 2013, at 58 years of age, the patient was referred to our department for endocrinological examination of short stature. The results of this examination showed impaired secretion of TSH on the thyrotropin-releasing hormone (TRH) stimulation test, impaired secretion of LH/FSH on the luteinizing hormone-releasing hormone (LH-RH) stimulation test, impaired secretion of GH on both arginine and GH-releasing peptide-**2** (GHRP-2) stimulation tests (Fig. 1), and an empty sella turcica with atrophy of the anterior pituitary gland on magnetic resonance imaging (MRI) (Fig. 2), but secretion of ACTH was retained on the corticotropin-releasing hormone (CRH) stimulation test (Fig. 3a). For this reason, GH-replacement therapy (0.075 mg/day of somatropin; genetic recombinant) was started. In addition, chronic kidney disease of unknown cause was identified (estimated glomerular filtration rate [eGFR], 31.9 mL/min/1.73 m^2^) (Table 1). At 63 years of age, hypoxia and hypercapnia were identified (partial pressure of carbon dioxide [pCO_2_], 59.6 mmHg; partial pressure of oxygen [pO_2_], 48.7 mmHg), and respiratory function testing confirmed a markedly restricted ventilation disorder, and both vital capacity (VC) and forced vital capacity (FVC) were significantly reduced (VC, 0.54 L; percentage predicted VC (%VC), 26.9%; FVC, 0.50 L), but percentage predicted forced expiratory volume in 1 s (%FEV_1.0_) was within normal limits (80.0%). Because the patient did not wish to receive home oxygen therapy (HOT) or respiratory rehabilitation, she was followed-up. However, HOT (0.5 L/min by nasal cannula) was started about 6 months later as her exertional dyspnea gradually worsened, and renal function also slowly decreased (eGFR, 25.0 mL/min/1.73 m^2^). Since there was no bronchiectasis or emphysema, pulmonary embolism and shunt disease were ruled out by the lung perfusion scan (technetium-99 m-labeled macro-aggregated albumin scintigraphy), and since there was weakness of proximal muscle without abnormal neurological findings, the patient was diagnosed with endocrine myopathy by both a respiratory physician and a neurologist. Furthermore, a nephrologist noted that there was no glomerulonephritis or hereditary kidney disease, and that there was unexplained renal atrophy. Around this time, frequent episodes of hypoglycemia occurred, and a low basal level of serum cortisol (6.38 μg/dL), a relatively low basal level of plasma ACTH (34.2 pg/dL), and a low 24-h urinary cortisol level (5.5 μg/day) were confirmed (Table 1). Delayed overreaction of plasma ACTH and failure of serum cortisol on the CRH stimulation test were confirmed (Fig. 3a, b), and a failure of plasma ACTH on the insulin tolerance test was confirmed (Fig. 3c). Furthermore, the cortisol response on the rapid ACTH stimulation test was slightly delayed and attenuated (Fig. 3d), and the prolonged ACTH stimulation test showed a sufficient increase in urinary free-cortisol levels (Fig. 3e). The patient was therefore diagnosed with hypothalamic secondary hypoadrenocorticism, and replacement therapy was started (prednisolone at 2.5 mg/day). At 65 years of age, the patient developed CO_2_ narcosis (pCO_2_, 80.1 mmHg, pO_2_, 76.0 mmHg) during bronchopneumonia treatment, and ventilatory function was: VC, 0.48 L; %VC, 27.1%; and FVC, 0.49 L. Noninvasive positive-pressure ventilation (NPPV) (2.0 L/min by mask) was started and continued at home.

**Table 1:** Laboratory findings

	At 58 years	At 63 years		
Biochemistry				
Alb	4.1	3.9	g/dL	[4.10–5.10]
Na	139	135	mmol/L	[138–145]
K	5.3	4.8	mmol/L	[3.6–4.8]
Cl	104	94	mmol/L	[101–108]
Ca	8.8	9.2	mg/dL	[8.8–10.1]
IP	5.0	3.0	mg/dL	[2.7–4.6]
CK	209	119	U/L	[41–153]
BUN	45.5	50.5	mg/dL	[8.0–20.0]
Cr	1.36	1.56	mg/dL	[0.46–0.79]
eGFR	31.9	26.8	mL/min/1.73 m^2^	
UA	5.0	7.9	mg/dL	[2.6–5.5]
CRP	0.26	0.06	mg/dL	[0.00–0.14]
Endocrinology				
FT3	2.8	NR	pg/mL	[2.48–4.14]
FT4	0.90	0.97	ng/dL	[0.76–1.65]
TSH	0.473	0.303	μU/mL	[0.541–4.261]
PRL	10.7	17.9	ng/mL	[3.12–15.39]
GH	0.13	1.07	ng/mL	[0.13–9.88]
IGF-1	28.0	19.0	ng/mL	[66.0–205.0]
LH	1.89	1.00	mIU/mL	[5.72–64.31]
FSH	4.05	2.69	mIU/mL	[< 157.79]
E2	< 10.0	< 5.0	pg/mL	[6.2–37.0]
ACTH 06:00	35.5	34.2	pg/mL	[7.2–63.3]
12:00	22.1	NR		
16:00	13.9	NR		
CORT 06:00	12.9	6.4	μg/dL	[6.2–18.0]
12:00	10.8	NR		
16:00	4.9	NR		
U-CORT	4.1	5.5	μg/day	[11.2–80.3]

Reference ranges are shown in brackets. *Alb* albumin, *IP* inorganic phospohorus, *CK* creatine kinase, *BUN* blood urea nitrogen, *Cr* creatinine, *eGFR* estimated glomerular filtration rate, *UA* uric acid, *CRP* C-reactive protein, *FT3* free triiodothyronine, *FT4* free thyroxine, *TSH* thyroid-stimulating hormone, *PRL* prolactin; *GH* growth hormone, *IGF-1* insulin-like growth factor 1, *LH* luteinizing hormone, *FSH* follicle-stimulating hormone, *E2* estradiol, *ACTH* adrenocorticotropic hormone, *CORT* cortisol, *U-CORT* urinary cortisol, *NR* no result

The patient was 129.4 cm tall and weighed 21.8 kg (body mass index, 13.2 kg/m^2^). Blood pressure was 110/57 mmHg, and heart rate was regular at 93 beats/min. She showed no mental retardation, and no pigmentation on the skin and oral mucosa. Her hair was thin, and no underarm or pubic hair was present. The Turner classification for the breasts was stage 1. Mild scoliosis was evident. Cardiopulmonary examination showed normal results, and no abnormal abdominal findings were identified.

## Discussion and conclusions

Patients with GHD can present with either an isolated deficiency or a combination of deficiencies of pituitary hormones [combined pituitary hormone deficiency (CPHD)] at any time from the neonatal period to adulthood. Additional endocrinopathies may develop in varying numbers and at various times [8]. It has been reported that additional hormone deficiencies were more frequent in the order of TSH, LH/FSH, ADH, and ACTH, and the median time interval from GHD diagnosis to the onset of other hormone deficiencies ranged from 1.9 years for TSH, to 2.4 years for ADH and ACTH, and 3.3 years for LH/FSH [9]. On the other hand, Otto et al. [2] reported that the most common deficiencies were LH/FSH deficiencies (38%), followed by TSH (31%), ACTH (12%), and ADH deficiencies (5%). In addition, they also reported that patients with various deficiencies presented at different times during follow-up: ADH deficiency at 3.1 ± 1 years; TSH deficiency at 7.5 ± 5.6 years; LH/FSH deficiencies at 8.3 ± 4 years; and ACTH deficiency at 9.3 ± 3.5 years [2]. Though these differences could be the result of differences in the age or follow-up period of the population studied, they may also be due to the variable endocrine phenotypes of CPHD patients [8]. In the majority of studies, the most frequent additional deficit was TSH deficiency, with the least frequent one being diabetes insipidus (DI); the prevalences of LH/FSH and ACTH deficiencies varied [8]. In addition, the following have been shown to be risk factors for progression from isolated GHD to CPHD: 1) severe GHD; 2) female sex; 3) organic GHD etiology; 4) longer follow-up; 5) genetic defects; 6) structural abnormalities of the forebrain and hypothalamo-pituitary region (ectopic posterior pituitary, absent pituitary stalk, small anterior pituitary, abnormal corpus callosum, septo-optic dysplasia, empty sella turcica, optic nerve hypoplasia, holoprosencephaly, etc.); 7) presence of extrapituitary malformations; and 8) delivery complications, breech delivery, and perinatal/neonatal adverse events [8]. This patient had an empty sella turcica, a 62-year period of follow-up, severe GHD, and female sex as risk factors. The interval from diagnosis of GHD to additional pituitary hormone deficiency was about 13 years for LH/FSH, about 47 years for TSH, and about 60 years for ACTH, although no DI was present. According to previous reports, the greatest age at onset of ACTH deficiency was in the 40s [10]; onset in the 60s is thus extremely rare.

The present patient also developed hypoxia and hypercapnia due to the markedly decreased VC. Adult patients with CPHD have been reported to show impairment of ventilatory function, and GH-replacement therapy can help restore it [4, 11, 12]. It has also been reported that GH-replacement therapy for GHD in adults results in increased maximal oxygen uptake, presumably due to increased respiratory muscle strength [13] and increased mean frequency of the surface electromyogram of the muscle fiber area in quadriceps [14]. However, only one report appears to have described severe respiratory failure requiring use of NPPV [12]. Furthermore, this patient had chronic kidney disease of unknown cause. In children with GHD, insulin growth factor (IGF)-1 activity has been reported to be significantly positively correlated with GFR [15]. Sohmiya et al. reported that chronic GH replacement improved progressive renal dysfunction in a patient with Sheehan’s syndrome associated with chronic renal failure [16]. On the other hand, since the action of IGF-1 is suppressed by an increase in IGF binding proteins, it has been reported that combined therapy with GH and IGF-1 is a reasonable treatment in chronic renal failure [17]. Checking GH secretion capacity may be important when the cause of respiratory or renal dysfunction is unclear. In the present case, the reason why GH replacement therapy did not improve the respiratory or renal disorder is considered to be because a sufficient amount of GH could not be administered due to the patient reporting a poor mood and the appearance of edema caused by increasing somatotropin. The prognosis of CPHD remains unclear due to very few reports with a long follow-up period. In particular, cases of CPHD that do not receive sufficient GH replacement therapy may have organ damage, as in the present case, and close attention is thus required.

One limitation of the present report was that genetic mutations in pituitary transcription factors (HESX1, PROP1, POU1F1, LHX3, LHX4, GLI2, and SOX3) were not confirmed [18]. Why progression of hypoadrenalism due to ACTH deficiency took so long also remains unclear, and further evaluations of this issue should be conducted in the future.

In conclusion, a rare case of a 65-year-old woman with childhood-onset GHD with panhypopituitarism, including late-onset secondary hypoadrenocorticism in her 60s, and severely impaired respiratory function and renal dysfunction, was presented. In GHD patients with risk factors for progression from isolated GHD to CPHD, lifelong endocrinological monitoring may be important.

## Data Availability

Not applicable.

## Abbreviations

GH: Growth hormone
GHD: Growth hormone deficiency
GFR: Glomerular filtration rate
eGFR: Estimated glomerular filtration rate
LH: Luteinizing hormone
FSH: Follicle-stimulating hormone
ACTH: Adrenocorticotropic hormone
TSH: Thyroid-stimulating hormone
TRH: Thyrotropin-releasing hormone
LH-RH: Luteinizing hormone-releasing hormone
GHRP-2: Growth hormone-releasing peptide-**2**
MRI: Magnetic resonance imaging
CRH: Corticotropin-releasing hormone
pCO_2_: Partial pressure of carbon dioxide
pO_2_: Partial pressure of oxygen
VC: Vital capacity
FVC: Forced vital capacity
%VC: Percentage predicted vital capacity
%FEV_1.0_: Percentage predicted forced expiratory volume in 1 s
HOT: Home oxygen therapy
NPPV: Noninvasive positive-pressure ventilation
CPHD: Combined pituitary hormone deficiency
ADH: Antidiuretic hormone
DI: Diabetes insipidus
